# The Impact of Psychiatric Patient Boarding in Emergency Departments

**DOI:** 10.1155/2012/360308

**Published:** 2012-07-22

**Authors:** B. A. Nicks, D. M. Manthey

**Affiliations:** Department of Emergency Medicine, Wake Forest University Health Sciences, Winston-Salem, NC 27157, USA

## Abstract

*Objectives*. Studies have demonstrated the adverse effects of emergency department (ED) boarding. This study examines the impact of resource utilization, throughput, and financial impact for psychiatric patients awaiting inpatient placement. *Methods*. The authors retrospectively studied all psychiatric and non-psychiatric adult admissions in an Academic Medical Center ED (*>*68,000 adult visits) from January 2007-2008. The main outcomes were ED length of stay (LOS) and associated reimbursement. *Results*. 1,438 patients were consulted to psychiatry with 505 (35.1%) requiring inpatient psychiatric care management. The mean psychiatric patient age was 42.5 years (SD 13.1 years), with 2.7 times more women than men. ED LOS was significantly longer for psychiatric admissions (1089 min, CI (1039–1140) versus 340 min, CI (304–375); *P* < 0.001) when compared to non-psychiatric admissions. The financial impact of psychiatric boarding accounted for a direct loss of ($1,198) compared to non-psychiatric admissions. Factoring the loss of bed turnover for waiting patients and opportunity cost due to loss of those patients, psychiatric patient boarding cost the department $2,264 per patient. *Conclusions*. Psychiatric patients awaiting inpatient placement remain in the ED 3.2 times longer than non-psychiatric patients, preventing 2.2 bed turnovers (additional patients) per psychiatric patient, and decreasing financial revenue.

## 1. Introduction

In recent years numerous studies have chronicled the adverse effects of emergency department boarding times on the prehospital, emergency department, and inpatient hospital financial and clinical outcomes in the USA and worldwide [1–13]. A small but increasing subset within this population are those patients presenting with psychiatric emergencies for which there is little published data. In the ever increasing challenges with access to health-care due to state and federal budget cuts, inpatient and outpatient psychiatric care options have noted substantial decreases. In some states, available inpatient capacity for primary psychiatric care has decreased by nearly 100% leading to increased queuing of those waiting for these resources and an increased burden on many emergency departments to board these patients while waiting for appropriate inpatient care options [14].

In addition, for the past 2 decades, emergency departments have seen increasing numbers of persons with psychiatric and substance abuse issues [14]; nationally, patients with mental health complaints account for 7% to 10% of ED visits [15, 16]. Despite accounting for a relatively small proportion of an emergency department's total census, these high-risk patients provide unique challenges for management. Substantial declines in mental health resources have contributed to increasing numbers of patients with mental health issues in emergency departments [14]. Inadequate outpatient psychiatric services for the uninsured and underinsured contribute to utilization of the emergency department as a primary source of psychiatric care. Reduced state and national funding and declining reimbursements resulted in inpatient unit closures and therefore prolonged ED stays [17]. Reduced availability of community-based referral options for follow-up care delays disposition and contributes to subsequent ED visits for similar complaints.

Patient “boarding,” the holding of a patient in an ED bed while awaiting an inpatient mental health bed, is a frequently reported occurrence. Studies cite an average of a 7-hour wait for a bed following the decision to admit, with an extended duration if transfer to an outside facility was required [15–17]. In a recent survey, numerous facilities reported instances of longer than 24 hours from bed request to patient transfer [17]. Prolonged ED stays are associated with increased risk of symptom exacerbation or elopement for patients with mental health/substance abuse issues. External stimuli from the busy emergency department can increase patient anxiety and agitation, which is potentially harmful for both patients and staff [15, 18]. Elopement from the emergency department prior to definitive screening and treatment can lead to increased risk of self-harm and suicide [18]. In addition, mental health patients in the emergency department contribute to other system issues such as increased ancillary resource utilization by safety attendants or security officers as a safety measure to protect staff and patients. This requirement leads to increased labor costs which have not been factored into this study. Patient care and customer relation issues can also arise as other patients are faced with the burden of additional wait time for emergency care. Poor clinical outcomes, evidenced as delays in care and increases in morbidity and mortality, have been directly associated with ED overcrowding and lack of available emergency beds and patients leaving without being seen [1–3, 18].

Patients with a psychiatric diagnosis, a substance abuse problem, or a dual diagnosis require specialized care to address their complex psychological, medical, and social needs. Optimally, these individuals are assessed and managed in safe, quiet, and calm areas, instead of the hurried, chaotic environment that is a characteristic of most emergency departments [18]. Generally, emergency physicians and nurses have modest clinical skills to manage these patients because most of their mental health training focused on initial diagnosis, care for related medical issues, and emergent interventions such as sedation or restraint. While some emergency departments have created positions such as a psychiatric or mental health liaison nurse or clinical nurse specialist to further address this concern, this alternative is not always feasible [19, 20]. Thus, the primary goal in most emergency departments is to keep the patients safe until they can be moved into a mental health unit or further stabilized and discharged home with an appropriate outpatient care plan [19, 20].

Acknowledging the varied adverse effects of prolonged emergency department (ED) boarding times on clinical and financial measures, this study sought to examine the impact on resources, throughput, and finances for all patients awaiting inpatient placement for emergent psychiatric conditions. Specifically, the study looked at the LOS for psychiatric patients as compared to nonpsychiatric adult inpatient admissions to floor or monitored beds (excluded ICU or step-down units), reimbursement for services provided during the ED care, and the opportunity cost of the impact on ED throughput.

## 2. Methods

This retrospective cohort analysis focused on all adult psychiatric admissions presenting to an Academic Emergency Department at a Level 1 Trauma Center and Tertiary Referral Center (>92,000 total patient visits; 68,000 adult visits), from January 2007 to January 2008. With 844 operational (885 licensed) patient beds including only 38 inpatient psychiatric beds, recurrent issues with psychiatric boarding in the 27 bed adult ED is common. The main outcomes were total ED LOS (arrival to transfer to inpatient bed or outside facility) and the physician and facility payment for services rendered. Data was collected from the electronic health record system within the institution using psychiatric consultation, department of admission or transfer as the identifier for those patients with a primary psychiatric admission diagnosis. Deidentified financial facility-based data were obtained for this population, as well as a cohort of general medicine patients requiring inpatient care on a floor or monitored bed; intensive care or step-down unit patients were excluded. This study was approved by the institutional review board.

The financial data in this study of ED psychiatric patient boarding was based on the facility payments received per admitted ED patient divided by the average total length of stay of all nonpsychiatric, non-ICU adult patients. This value was used to identify the average hourly ED bed payment. This hourly amount was then multiplied by the total LOS for the psychiatric patients to identify what the total payment should be for psychiatric patients based on this length of stay. Then this value was subtracted from the facility payments for the nonpsychiatric cohort to identify the potential payment loss. Secondary assessment of the average ED physician payments for the same cohort was obtained. This was then applied to the potential missed patients being seen during 12 hours per day which represent the daily duration that ED patients are awaiting an unavailable bed due to psychiatric patient boarding at this facility. The potential payment losses due to decreased bed turnover and the payment differences were combined to identify the psychiatric patient boarding losses per patient.

## 3. Results

Table 1 summarizes the demographic and length of stay data from the psychiatric and nonpsychiatric cohorts. During the study period, 1,438 patients were consulted to psychiatry with 505 (35.1%) requiring inpatient care management for their psychiatric condition. The mean age of the psychiatric patients was 43 years (SD 13.1 years), with 2.7 times more women than men compared to 52 years and a slight female predominance in the nonpsychiatric group. The psychiatric group was 50% white, non-Hispanic and 42% African American compared to 54% and 39% respectively in the nonpsychiatric group.

Table 1 also shows the length of stay data for the 505 psychiatric and 18768 nonpsychiatric admissions. The total ED LOS was significantly longer for psychiatric patient admissions (1089 min, CI [1039–1140] versus 340 min, CI [294–372]; *P* < 0.001) when compared to nonpsychiatric admissions. The duration from consultation to admission or transfer was also significantly longer for psychiatric patients (1017 min, CI [957–1082] versus 178 min, CI [159–201]; *P* < 0.001).

The hourly payment for an ED bed was calculated to be $99.50. When applied to the total LOS for the psychiatric patients less the facility average payment per admitted patient, the facility payment loss for each admitted or transferred psychiatric patient was $1,198. This was then applied to the potential missed patients being seen assuming patients are awaiting an unavailable bed in the ED due to psychiatric patient boarding. Factoring the financial factors associated with the loss of bed turnover for waiting patients, psychiatric patient boarding awaiting inpatient placement cost the department $2,264 per patient.

## 4. Discussion

Overcrowding in the ED is not just an inconvenience of elongated wait times, hallway boarding of patients, and frequently HIPAA privacy failures; it is evidence of a health care system quality failure. As has been clearly delineated in recent literature, overcrowded hospital and emergency department (ED) conditions are associated with an increased risk of death or disability [1–5], an increased door-to-needle time delay for treatment of patients with suspected acute myocardial infarction and poorer performance on pneumonia quality of care measures [6–8]. A recent meta-analysis also found overcrowding to be associated with increased transport delays, ambulance diversion, patients leaving without being seen, and medication errors, among other problems [10]. Each of these issues directly identify the impact on patient care but when discussed at the administrative level many improvement projects are limited by short-term financial discussions [11–13].

The aforementioned issues related to boarding are further exacerbated in the psychiatric population due to decreasing inpatient care beds and bed availability. While a small subset of the total admitted population in the study (2.6%), the total length of stay (1089 min; 18.2 hours) for psychiatric patients was significantly greater than the nonpsychiatric cohort (340 min; 5.7 hours). The duration from time of consultation to admission for these cohorts was (1017 versus 178 minutes, respectively). This identifies the examination time period for psychiatric patients was shorter by approximately 100 minutes. Uniform in the admission process for psychiatric patients is a preset laboratory screening panel, but unlike the nonpsychiatric subset, imaging was not routinely obtained and likely accounts for the examination time difference. A recent study by Weiss et al. found similar length of stay for admitted psychiatric patients and also found that the need for hospitalization, older age, intoxication, and insurance status were associated with increased length of stay [15].

The psychiatric cohort was younger (median age, 43 versus 52) and more predominately female (63 versus 57%). While there were minimal differences in racial categories between cohorts, the lack of insurance was higher in the psychiatric cohort (32 versus 24%). Given the median age and the sex breakdown of the cohorts, this was not seen to be a likely contributing factor in increased length of stay. However, the higher uninsured rate of the psychiatric may be associated with the increased length of stay [15].

The financial impact associated with admitted psychiatric patients within our patient population and payor mix was associated with a 40% decrease in average physician reimbursement when compared to the nonpsychiatric cohort. This aside, the increase in total length of stay per admitted psychiatric patient prevents the ED from caring for an additional 2.2 patients and growing due to resource limitations within the state. Applying the average financials for each ED patient that would otherwise be cared for, the impact from each psychiatric boarded patient represents a loss to the system of approximately $2,400. Certainly, if greater than 20% of your ED capacity is being occupied with psychiatric boarded patients, the financial impact would be far greater than the conservative estimates presented as it would likely have downstream impact on system patient flow, patient satisfaction, potential shift in payor mix if seen as an undesirable place to receive care, ability to provide exceptional care, meeting all core measure expectations, and perceived community benefit from the ED. However, if even a fraction of the financial loss demonstrated were placed in to resource development and solution generation for this patient population, the net gain may be profound.

It is also important to recognize that while this was a single center study, in a survey of 328 ED Medical Directors in the United States, 79.2% report routine psychiatric patient boarding with 35.1% boarding greater than 1 patient per day and 38.9% boarding for between 8 and 24 hours. This survey sited lack of accepting transferring facility (19.9%), inability to transfer to an accepting facility due to bed availability (19.5%), and lack of in-house inpatient psychiatric beds (16.5%) as the most common reasons for extended ED length of stay [21]. Recognizing one of the root causes for psychiatric boarding is lack of available care options is an essential part of initiating change. This may reflect the needs to improve the input, throughput, and outpatient care follow-up psychiatric inpatients and thereby creating available capacity. Further exploration of psychiatric care approaches to reduce hospitalization by identifying transition of care alternatives may expedite the transition to outpatient care, and subsequently have a positive effect on ED length of stay [19, 20].

## 5. Limitations

It is important to recognize that the data presented represents an experience from a single, large academic center with an inpatient psychiatric care unit and therefore is not uniformly generalizable. For those EDs that have an external but associated facility, boarding may not currently be an issue, just the medical clearance of this patient population. Other EDs may lack attached or affiliated inpatient psychiatric care options and therefore may face very similar issues, perhaps greater, depending on transfer center availability.

The data in this study was obtained from the electronic health record, rather than prospective data collection, which may not be a perfect reflection of the exact time allotment for each patient, but remains representative of the patient data as a whole. During the study period, the ED maintained a period of full capacity for all but an average 8 hour period from approximately 3 am–11 am during which time the financial impact of patient queuing or increased LWBS would not be appreciated. However, the associated decreased payments due to psychiatric boarding or elongated LOS would still equate to a loss but would not include a loss related to an increased LWBS population. It is also important to note that the cost related to increased resource utilization for security throughout the patient stay, or the associated increase in staffing number required, especially in the overnight hours when total patient volume otherwise decreases, is not included in this calculation but would only increase the associated financial impact.

Further assessment considering insurance status at time of presentation might further delineate the anticipated longer boarding for the uninsured yet; a large amount of uncompensated care is provided within the study facility which may not be the case, elsewhere. Regardless, in this current economic climate, available resources for this unique patient population are limited and likely to worsen into the foreseeable future even, with pending health care reform. Our data supports the premise that overcrowding is a complex process, but that growing number of psychiatric boarding patients not only magnifies this process but also has a notable financial impact on the facility. If not already noted, this should be a signal to those planning the future of health care—whether those developing APCs, medical home models, or further governmental developed social care networks—that the current resources for psychiatric patients are limited and the proverbial “safety net”—the ED—remains at the breaking point.

## 6. Conclusions

Psychiatric patients awaiting inpatient placement remain in the ED 3.2 times longer than nonpsychiatric ED patients. Longer length of stays of psychiatric patients prevent 2.2 bed turnovers (or additional patients seen) per psychiatric patient awaiting inpatient care. The loss of payments due to boarding psychiatric patients awaiting inpatient bed placement is an approximate $2,250. While the exact data may not be consistent between facilities and states due to resources and patient population, if appropriate access to psychiatric care is available, the improved efficiencies of care (and associated financials) would help foster greater financial support for further psychiatric care while improving ED capabilities. This may include improved medical home models that include psychiatric assessment and direct care disposition to inpatient facilities, transition of care alternatives besides current inpatient care models or perhaps improvement in baseline psychiatric care to decrease the need for emergent ED interventions.

## Figures and Tables

**Table 1:** Demographic and length of stay data of the patient cohorts.

	Psychiatric	Nonpsychiatric	Significance (*P*)
Total admissions	505 (2.6%)	18768 (97.4%)	
Age in years, median	43	52	*P* < 0.05
Sex (%)			
Male	37	43	*P* < 0.05
Female	63	57	
Racial categories (%)			
White	50	54	
African-American	42	39	^ ∗∗^NS
Hispanic	6	5	
Asian	0.3	0.4	
Uninsured (%)	32	24	*P* < 0.05
Total length of stay (min)	1089	340	*P* < 0.001
Duration from Consultation to Admission (min)	1017	178	*P* < 0.001

Key: ^∗∗^NS: not statistically significant, min: minute.
